# Supplementary material for: Neuronal hyperexcitability is a DLK-dependent trigger of herpes simplex virus reactivation that can be induced by IL-1
Source: eLife. 2020 Dec 22;9:e58037. doi: 10.7554/eLife.58037 (PMC7773336; doi:10.7554/eLife.58037)
Supplement: Supplementary file 1. — Cell Body Score for Neuronal Health and Degeneration IndexScoring system used to determine neuronal health based on morphology of the soma following treatment with compounds used in this study. Table 2. Axon Score for Neuronal Health and Degeneration IndexScoring system used to determine neuronal health based on morphology of the axons following treatment with compounds used in this study. [file elife-58037-supp1.docx]

Supplemental Materials and Methods Tables

Table S1: Compounds Used and Concentrations

| Compound | Supplier | Identifier | Concentration |
| --- | --- | --- | --- |
| Acycloguanosine | Millipore Sigma | A4669 | 10 µM, 50 µM |
| FUDR | Millipore Sigma | F-0503 | 20 µM |
| Uridine | Millipore Sigma | U-3003 | 20 µM |
| SP600125 | Millipore Sigma | S5567 | 20 µM |
| GNE-3511 | Millipore Sigma | 533168 | 4 µM |
| GSK-J4 | Millipore Sigma | SML0701 | 2 µM |
| L-Glutamic Acid | Millipore Sigma | G5638 | 3.7 µg/mL |
| Forskolin | Tocris | 1099 | 60 µM |
| LY 294002 | Tocris | 1130 | 20 µM |
| 666-15 | Tocris | 5661 | 2 µM |
| SQ 22,536 | Tocris | 1435 | 50 µM |
| KT 5720 | Tocris | 1288 | 3 µM |
| TEA | Tocris | 3068 | 10 mM |
| CsCl | Tocris | 4739 | 3 mM |
| OG-L002 | Tocris | 6244 | 30 µM |
| S2101 | Tocris | 5714 | 20 µM |
| Tetrodotoxin | Tocris | 1069 | 1 µM |
| ESI-09 | Tocris | 4773 | 10 µM |
| ZD 7288 | Cayman | 15228 | 20 µM |
| 8-bromo-cyclic AMP | Cayman | 14431 | 125 µM |
| NGF 2.5S | Alomone Labs | N-100 | 50 ng/mL |
| Primocin | Invivogen | ant-pm-1 | 100 µg/mL |
| Aphidicolin  IL-1β | AG Scientific  Shenandoah Bio. | A-1026  100-167 | 3.3 µg/mL  30ng/mL |
| WAY-150138  Anti-Mouse IL-1R  Fura-2, AM  Hoescht | Pfizer/Gift  Leinco Technologies  Thermo Fisher  Thermo | NA  I-736  F1221  62249 | 10 µg/mL  2 µg/mL  5 µM  2uM |

Table S2: Primers Used for RT-qPCR

| Primer | Sequence 5’ to 3’ |  |
| --- | --- | --- |
| mGAP 1SF | CAT GGC CTT CCG TGT GTT CCT A |  |
| mGAP 1SR | GCG GCA CGT CAG ATC CA |  |
| ICP27 F | GCA TCC TTC GTG TTT GTC ATT CTG |  |
| ICP27 R | GCA TCT TCT CTC CGA CCC CG |  |
| ICP8 1SF | GGA GGT GCA CCG CAT ACC |  |
| ICP8 1SR | GGC TAA AAT CCG GCA TGA AC |  |
| ICP4 F | TGC TGC TGC TGT CCA CGC |  |
| ICP4 R | CGG TGT TGA CCA CGA TGA GCC |  |
| UL30 F | CGC GCT TGG CGG GTA TTA ACA T |  |
| UL30 R | TGG GTG TCC GGC AGA ATA AAG C |  |
| UL48 F | TGC TCG CGA ATG TGG TTT AG |  |
| UL48 R | CTG TTC CAG CCC TTC ATG TT |  |
| gC F | GAG TTT GTC TGG TTC GAG GAC |  |
| gC R | ACG GTA GAG ACT GTG GTG AA |  |

Table S3: Antibodies Used for Western Blotting and Concentrations

| Antibody | Supplier | Identifier | Concentration |
| --- | --- | --- | --- |
| Rb Phospho-Akt (S473) | CST | 4060 | 1:500 |
| Rb Akt (pan) | CST | C67E7 | 1:1000 |
| Rb Phospho-c-Jun (S73) | CST | 3270 | 1:500 |
| Anti-DLK/MAP3K12 | Thermo Fisher | PA5-32173 | 1:500 |
| Ms Monoclonal α-Tubulin | Millipore Sigma | T9026 | 1:2500 |
| HRP Goat Anti-Rabbit IgG Antibody (Peroxidase) | Vector | PI-1000 | 1:10000 |
| HRP Horse Anti-Mouse IgG Antibody (Peroxidase) | Vector  Thermo Fisher | PI-2000  PA5-32173 | 1:10000  1:500 |

Table S4: Antibodies Used for Immunofluorescence and Concentrations

| Antibody | Supplier | Identifier | Concentration |
| --- | --- | --- | --- |
| Rb H3K9me3S10P | Abcam | ab5819 | 1:250 |
| Ch Beta-III Tubulin  Ms γH2A.X  Ms c-Fos | Millipore sigma  CST  Novus | AB9354  80312S  NB110-75039 | 1:1000  1:100  1:125 |
| F(ab’)2 Goat anti Mouse IgG (H+L) Alexa Fluor® 647 | Thermo Fisher | A21237 | 1:1000 |
| F(ab’) Goat anti Rabbit IgG (H+L) Alexa Fluor® 555 | Thermo Fisher | A21425 | 1:1000 |
| Goat anti Chicken IgY (H+L) Alexa Fluor® 647  Goat Anti-Chicken IgY H&L (Alexa Fluor® 488) preabsorbed  F(ab’)2 Goat anti-Rabbit IgG (H+L) Alexa Fluor® 488) | abcam  abcam  Thermo Fisher | ab150175  ab150173  B40922 | 1:1000  1:1000  1:1000 |

Table S5: Cell Body Score for Neuronal Health and Degeneration Index

| Score | Description |
| --- | --- |
| 0 | Large, phase bright cell bodies. Clear with no fragmentation or vesiculation. |
| 1 | Small, phase bright cell bodies. Clear with no fragmentation or vesiculation. |
| 2 | Cell bodies do not have fragmentation but are not phase bright. Sometimes appear transparent. |
| 3 | Cell bodies with fragmentation but few dead neurons or corpses. |
| 4 | Cell bodies with fragmentation with many corpses present and neurons starting to detach. |
| 5 | Complete cell death. Neurons detached. |

Table S6: Axon Score for Neuronal Health and Degeneration Index

| Score | Description |
| --- | --- |
| 0 | Axons totally smooth with no blebbing or fragmentation. Branched and form a spider web-like network. |
| 1 | Axons smooth but grow straight. |
| 2 | Blebbing on the axons but no apparent fragmentation. |
| 3 | Fragmentation starting to appear in <50% of the neurons. |
| 4 | Fragmentation in >50% of the neurons. |
| 5 | No axons remaining. |
